# Ultrasound characteristics of thyroid nodules facilitate interpretation of the malignant risk of Bethesda system III/IV thyroid nodules and inform therapeutic schedule

**DOI:** 10.1002/dc.24248

**Published:** 2019-06-18

**Authors:** Fu Li, Denghua Pan, Yuquan Wu, Jinbo Peng, Qing Li, Xiaolong Gui, Wei Ma, Hong Yang, Yun He, Junqiang Chen

**Affiliations:** ^1^ Department of Gastrointestinal Surgery First Affiliated Hospital of Guangxi Medical University Nanning Guangxi Zhuang Autonomous Region People's Republic of China; ^2^ Department of Ultrasonography First Affiliated Hospital of Guangxi Medical University Nanning Guangxi Zhuang Autonomous Region People's Republic of China; ^3^ Department of Pathology First Affiliated Hospital of Guangxi Medical University Nanning Guangxi Zhuang Autonomous Region People's Republic of China

**Keywords:** Bethesda system III/IV, thyroid nodule, ultrasound characteristics, US‐FNA

## Abstract

**Background:**

This study was designed to explore whether ultrasound of thyroid nodules facilitates the interpretation of the malignant risk of Bethesda III/IV thyroid nodules to inform further therapies.

**Methods:**

We reviewed patient records in which the results of ultrasound‐guided fine‐needle aspiration (US‐FNA) were classified by the Bethesda III/IV in our institution between January 2016 and June 2018. Studies were retrieved from PubMed, Cochrane Central Register of Controlled Trials, ISI Web of Science, Science Direct, Wiley Online Library, EMBASE, China National Knowledge Infrastructure, WanFang, and Chinese VIP. The odds ratio (OR) was used to measure associations between risk factors and thyroid nodule malignancy.

**Results:**

Fifty‐nine cases of Bethesda III/IV with corresponding surgeries were included, and the malignancy risk was 54.2%. Meta‐analysis revealed irregular borders, solitary nodules, hypoechogenicity, microcalcifications, and being taller than wide, all of which increased the malignancy risk of thyroid nodules. Combined ORs for these factors were 4.08 (95% CI: 2.34‐7.14, *P* < .001), 2.18 (95% CI: 1.39‐3.42, *P* = .001), 2.02 (95% CI: 1.35‐3.01, *P* = .001), 3.21 (95% CI: 2.26‐4.56, *P* < .001), and 4.35 (95% CI: 3.07‐6.15, *P* < .001), respectively.

**Conclusion:**

As the risk of malignancy for papillary thyroid carcinoma (PTC) is high, when any one of the five ultrasound features of malignancy were confirmed, repeated FNA is recommended to confirm PTC‐type malignancy, even though nodules were Bethesda III/IV classification. However, repeated FNA should be avoided when none of these ultrasound features are identified because repeated FNA does not contribute to identifying non‐PTC type malignancies, such as follicular thyroid carcinoma and poorly differentiated thyroid carcinoma.

## 1 INTRODUCTION

Thyroid nodules are clinically common; however, the occurrence of malignancy in thyroid nodules remains relatively low, ranging between 5% and 7%.^1^, ^2^ Ultrasound‐guided fine‐needle aspiration (US‐FNA) is a useful test for the evaluation of thyroid nodules that has been widely applied as the primary diagnostic procedure.^3^ FNA results are standardized by the Bethesda System for Reporting Thyroid Cytopathology, which promotes effective communication between clinicians^2^, ^4^ and are divided into six types. Benign nodules are classified as Bethesda II, suspicious nodules are classified as Bethesda V, and malignant nodules are classified as Bethesda VI. Undetermined nodules are classified as either Bethesda III (atypia of undetermined significance or follicular lesion of undetermined significance [AUS/FLUS]) or Bethesda IV (follicular neoplasm or suspicious for follicular neoplasm [FN/SFN]). Thyroid nodules classified as Bethesda II, V, and VI have prescribed management strategies; in contrast, optimal management strategies for uncertain nodules (Bethesda III and IV) remain unclear. The risk of malignancy for Bethesda III is estimated at 5%‐15%,^2^ and 10%‐40% of Bethesda IV nodules are malignant.^3^ Due to the large range of malignancy risk, repeated FNA or surgery is recommended, and more than half have a determined diagnostic result.^5^, ^6^, ^7^ Approximately 15.6%‐48.6% of nodules remain AUS/FLUS upon repeat FNA,^5^, ^6^, ^8^ indicating a need to assess the risk of malignancy in Bethesda system III/IV nodules.

Ultrasound is the preferred method to detect thyroid nodules, and ultrasound factors, such as hypoechogenicity, irregular shape, being taller than wide and microcalcifications, are considered to be features of malignant nodules. Some studies have demonstrated the use of ultrasound to predict the risk of malignancy in Bethesda III and IV thyroid nodules during the initial US‐FNA.^9^, ^10^, ^11^, ^12^ However, one study demonstrated that the thyroid ultrasound risk stratification system has poor specificity and accuracy for classifying indeterminate lesions.^13^ Another study reported that age and gender, but not ultrasound characteristics, influence the decision to perform surgery in AUS/FLUS patients.^14^ Since then, no clear guidelines have been established to manage Bethesda III and IV nodules. The purpose of this study was to explore whether ultrasound characteristics of thyroid nodules may facilitate clinicians interpreting the risk of malignancy for Bethesda system III/IV thyroid nodules to inform choices for future therapies.

## 2 MATERIALS AND METHODS

### 2.1 Sonographic evaluation

This study was approved by the Ethics Committee of the First Affiliated Hospital of Guangxi Medical University. All US‐FNA reports and histopathological diagnoses from 1147 patients who were admitted and received surgery from thyroid nodules in the First Affiliated Hospital of Guangxi Medical University from January 2016 to June 2018 were retrospectively reviewed. US‐FNA results were described using the Bethesda System for Reporting Thyroid Cytology (TBSRTC), and patients defined as Bethesda system III/IV were enrolled in our study. Patient thyroid nodules were measured using LOGIE9 (GE Healthcare, Wauwatosa, Wisconsin). The transducer was a 5‐12 MHz linear transducer. The following ultrasound imaging features of nodules were evaluated: size, shape, being taller than wide, solitary nodules, echogenicity, and calcification. Malignant imaging features included irregular shape, hypoechogenicity, being taller than wide, and microcalcification.

### 2.2 Search strategy

A literature search was performed to identify all articles related to ultrasound parameters in Bethesda system III/IV nodules in the following databases: PubMed, Cochrane Central Register of Controlled Trials, ISI Web of Science, Science Direct, Wiley Online Library, EMBASE, China National Knowledge Infrastructure, WanFang, and Chinese VIP from inception to August 2018 using the keywords: “thyroid,” “Bethesda system,” “The Bethesda System for Reporting Thyroid Cytology “TBSRTC,” “fine‐needle aspiration,” “FNA,” and “FNAC.” Inclusion criteria were studies using ultrasound to estimate the malignancy of Bethesda III/IV nodules in the initial US‐FNA. Reviews and references were also evaluated. Reviews, abstracts, letters, and duplicate data were removed. No language restrictions were applied.

Two authors (Fu Li and Denghua Pan) independently performed the literature search and data screening. When there was controversy, the disagreement was resolved by a third reviewer (Yun He).

### 2.3 Data extraction

Two investigators (Fu Li and Denghua Pan) independently extracted data from eligible studies. The following characteristics were extracted: first author's name, year of publication, country, and number of patients. To evaluate risk factors associated with thyroid cancer, the following ultrasound parameters were extracted: irregular borders, solitary nodule, hypoechogenicity, microcalcifications, and being taller than wide.

### 2.4 Statistical analysis

Two independent *t*‐tests and chi‐square test were applied to analyze differences between benign and malignant groups using SPSS22.0 (SPSS Inc., Chicago, Illinois). For meta‐analysis, the odds ratio (OR) was used to explore associations between risk factors and thyroid nodule malignancy. OR > 1 indicates that risk factors were more likely to correlate with the malignancy of thyroid nodules. For heterogeneity analysis, chi‐squared test was used to assess heterogeneity among studies. *P* < .05 was considered to represent significant heterogeneity, and then a fixed effect model was used. Otherwise, a random effects model was used. Publication bias was measured using funnel plots. Meta‐analysis was conducted in STATA12.0 (STATA Corp., College, Texas). *P* < .05 was considered statistically significant.

## 3 RESULTS

### 3.1 Clinical and ultrasonographic characteristics of Bethesda system III/IV nodules

From June 2016 to 2018, there were 1147 cases of FNAB performed at our institution. Eighty‐nine patients were diagnosed as Bethesda system III thyroid nodules and twenty‐nine patients were diagnosed as Bethesda system IV thyroid nodules and. The resection rate of Bethesda system III was 43.8% (39/89), the resection rate of Bethesda system IV was 69.0% (20/29). Among 59 patients, 46 were female and 13 were male. Cases were classified into benign and malignant groups based on final histopathologic diagnosis. Table 1 shows clinical and ultrasonographic characteristics of Bethesda system III/IV nodules. In the malignant group, 30 cases were papillary thyroid carcinoma (PTC), one was follicular thyroid carcinoma (FTC), and other was medullary thyroid carcinoma (MTC). The average ages of patients in the benign group were 46.4 and 42.0 years old in malignant group, respectively. No significant difference was observed with respect to age between benign and malignant groups (*P* > .05). In the benign group, average nodule size was 2.51 cm, and in the malignant group, mean nodule size was 1.44 cm, which were significantly different in two groups (*P* = .019). Additionally, there was a significant difference between benign and malignant groups with respect to patient sex (*P* = .013) in thyroid nodules. In the malignant groups of thyroid nodules, the parameters of irregular borders, solitary nodules, hypoechogenicity, microcalcifications and being taller than wide were more common (all *P* < .05). We also compared clinical ultrasound and ultrasonographic characteristics between PTC and benign thyroid nodules (Table 2). The mean age of PTC cases was 41.27 years old, and the average age of the benign group was 46.41 years old, with no significant difference between the two group (*P* = .119). Average size in the benign group was larger than in the PTC group (*P* = .023). In addition, there were significant differences in sex, irregular borders, solitary nodules, hypoechogenicity, microcalcifications, and being taller than wide between PTC and benign groups (all *P* < .05).

**Table 1:** Clinical and ultrasonographic characteristics of Bethesda category III/IV

Parameters		Benign (n = 27)	Malignant (n = 32)	*P* value
Age		46.41 ± 12.91	42.00 ± 11.65	.174
Sex	Female	25 (92.6%)	21 (65.6%)	.013
	Male	2 (7.4%)	11 (34.4%)	
Size (cm)		2.51 ± 1.62	1.44 ± 1.76	.019
Irregular borders	Yes	6 (22.2%)	26 (81.3%)	<.001
	No	21 (77.8%)	6 (18.7%)	
Solitary nodule	Yes	13 (48.1%)	30 (93.8%)	<.001
	No	14 (51.9%)	2 (6.2%)	
Hypoechoic	Yes	7 (25.9%)	30 (93.8%)	<.001
	No	20 (74.1%)	2 (6.2%)	
Microcalcifications	Yes	0 (0.0%)	17 (53.1%)	<.001
	No	27 (100.0%)	15 (46.9%)	
Being taller than wide	Yes	1 (3.7%)	8 (25.0%)	.023
	No	26 (96.3%)	24 (75.0%)	

**Table 2:** Clinical and ultrasonographic characteristics between PTC and benign thyroid nodules

		Benign (n = 27)	PTC (n = 30)	*P* value
Age		46.41 ± 12.91	41.27 ± 11.60	.119
Sex	Female	25	20	.023
	Male	2	10	
Size (cm)		2.51 ± 1.62	1.27 ± 1.68	.006
Irregular borders	Yes	6	25	<.0001
	No	21	5	
Solitary nodule	Yes	13	29	<.0001
	No	14	1	
Hypoechoic	Yes	7	29	<.0001
	No	20	2	
Microcalcifications	Yes	0	17	<.0001
	No	27	13	
Being taller than wide	Yes	1	8	.018
	No	26	22	

Abbreviation: PTC, papillary thyroid carcinoma.

### 3.2 Characteristics of studies included in this meta‐analysis

**Figure 1:**
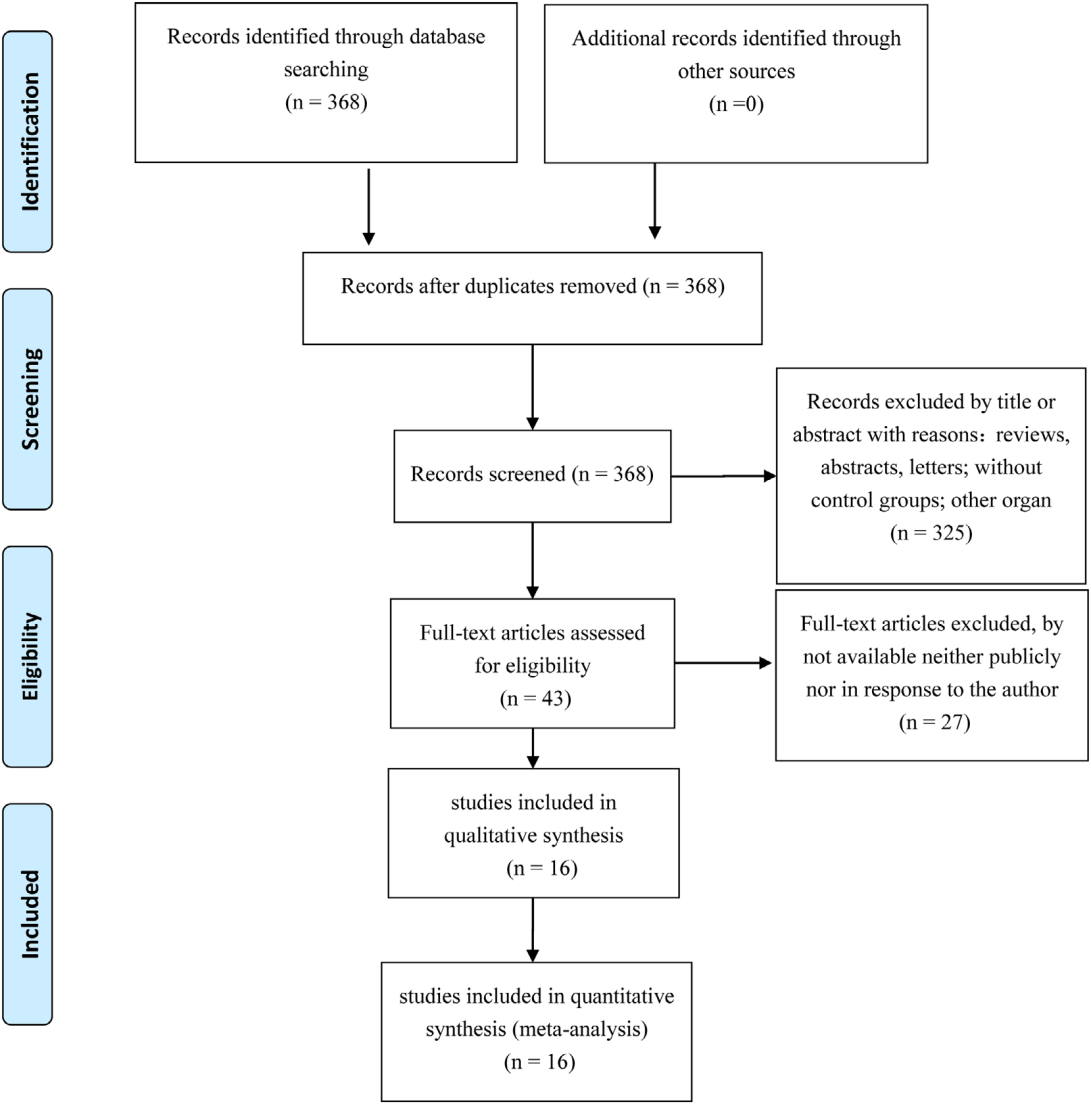
Flow diagram of this study. A total of 368 publications and microarray datasets were initially obtained. According to the exclusion criteria, only 16 were subsequently included in this study [Color figure can be viewed at wileyonlinelibrary.com]

**Figure 2:**
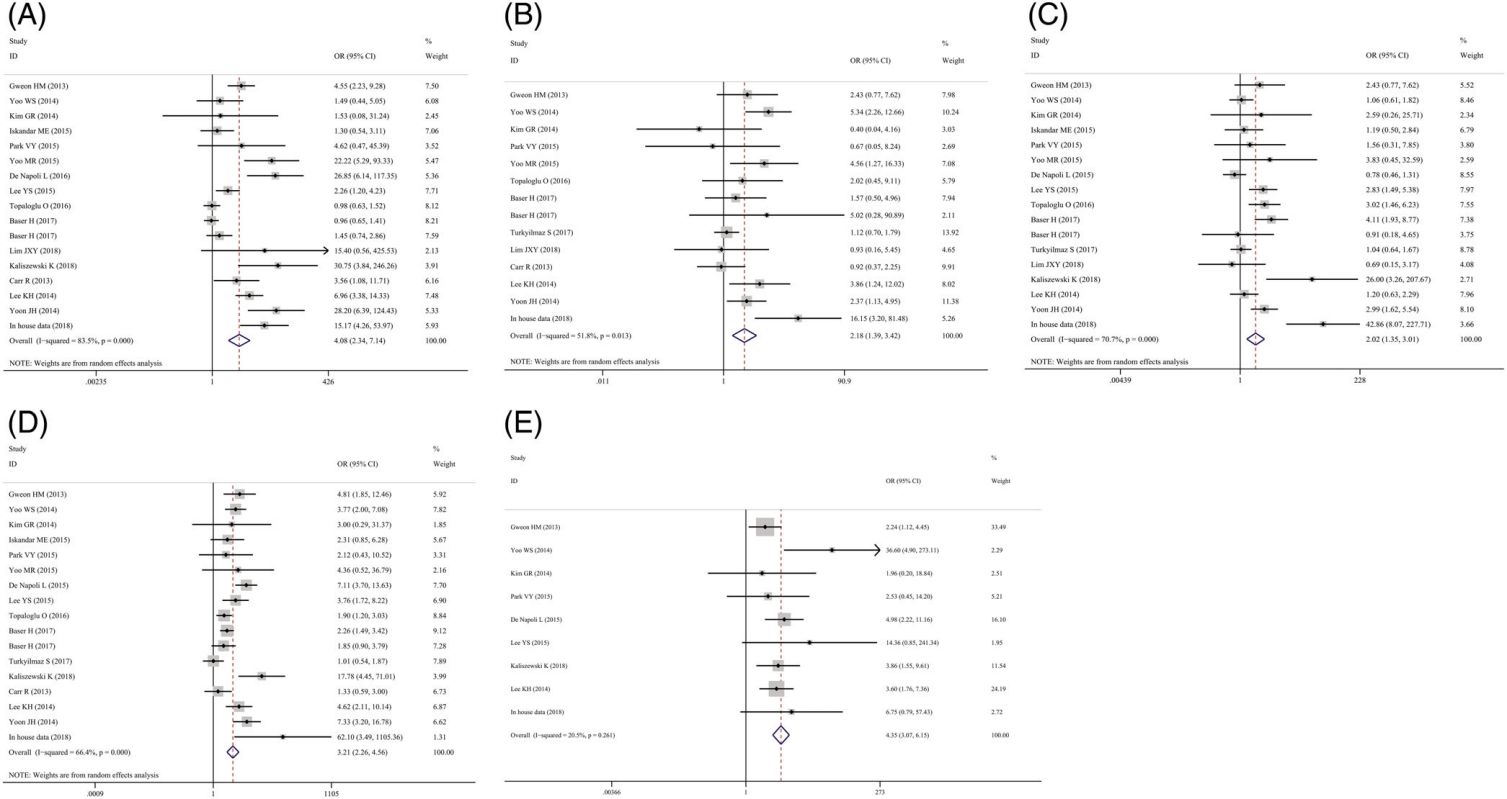
Meta‐analysis of included studies assessing the association between risk factors and thyroid cancer. A, Irregular borders. B, Solitary nodule. C, Hypoechoic. D, Microcalcifications. E, Being taller than wide [Color figure can be viewed at wileyonlinelibrary.com]

Figure 1 shows the procedures of this study, which screened 368 studies. After reading full texts of the studies, 16 publications were included in the meta‐analysis.^9^, ^15^, ^16^, ^17^, ^18^, ^19^, ^20^, ^21^, ^22^, ^23^, ^24^, ^25^, ^26^, ^27^, ^28^, ^29^ Table 3 shows the characteristics included studies. All studies had sufficient data to calculate the associations between risk factors and nodules. This meta‐analysis observed significant associations between irregular borders, solitary nodules, hypoechogenicity, and microcalcifications and nodules that are being taller than wide or malignant. Combined ORs were 4.08 (95% CI: 2.34‐7.14, *P* < .001; Figure 2A), 2.18 (95% CI:1.39‐3.42, *P* = .001; Figure 2B), 2.02 (95% CI:1.35‐3.01, *P* = .001; Figure 2C), 3.21 (95% CI: 2.26‐4.56, *P* < .001; Figure 2D), and 4.35 (95% CI: 3.07‐6.15, *P* < .001), respectively, (Figure 2E; Table 4).

**Table 3:** Characteristics of included studies

First author	Year	Country	Bethesda category
Gweon et al^9^	2013	South Korea	III
Carr et al^15^	2013	American	IIIS
Yoo et al^16^	2014	Korea	III
Lee et al^17^	2014	Korea	AUS
Yoon et al^18^	2014	Seoul Korea	III
Kim et al^19^	2014	Korea	AUS
Iskandar et al^20^	2015	American	III/IV
Park et al^21^	2015	Seoul, Republic of Korea	III
Yoo et al^22^	2015	Seoul, Republic of Korea	III
De Napoli et al^23^	2016	Italy	IV
Lee et al^24^	2015	Seoul, Korea	III
Topaloglu et al^25^	2016	Turkey	III
Baser et al^26^	2017	Turkey	FLUS
Turkyilmaz et al^27^	2017	Turkey	III
Lim et al^28^	2018	Singapore	III
Kaliszewski et al^29^	2018	Poland	III

Abbreviation: AUS/FLUS, atypia of undetermined significance or follicular lesion of undetermined significance.

**Table 4:** Risk factors associated with thyroid cancer

Risk factor	Malignant total	Event	Benign total	Event	Heterogeneity *I* ^2^	*P* value	Meta‐analysis model	Odds ratio (95% confidence interval)	*P* value
Irregular borders	1092	513	1894	576	83.50%	<.001	random effect analysis	4.08 (2.34‐7.14)	<.001
Solitary nodule	940	824	1695	1399	51.80%	.013	random effect analysis	2.18 (1.39‐3.42)	.001
Hypoechoic	1163	558	2070	665	70.70%	<.001	random effect analysis	2.02 (1.35‐3.01)	.001
Microcalcifications	1208	438	2094	353	66.40%	<.001	random effect analysis	3.21 (2.26‐4.56)	<.001
Being taller than wide	565	174	766	79	20.50%	.261	fixed effect analysis	4.35 (3.07‐6.15)	<.001

### 3.3 Publication bias

**Figure 3:**
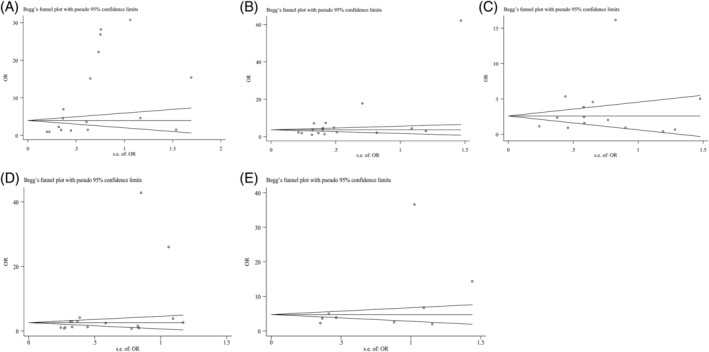
Funnel plots were used to estimate potential publication bias. A, Irregular borders. B, Microcalcifications. C, Solitary nodule. D, Hypoechoic. E, Being taller than wide. Begg's method was applied

Begg's test was performed to measure publication bias of this meta‐analysis. Publication bias was found in groups with irregular borders (Figure 3A, *P* = .003) and microcalcifications (Figure 3B, *P* = .019). No publication bias was present for solitary nodules (Figure 3C, *P* = .235), hypoechogenicity (Figure 3D, *P* = .055), and those that are being taller than wide (Figure 3E, *P* = .173).

### 3.4 Discussion

Ultrasound is the preferred method to detect thyroid nodules, and US‐FNA is a helpful procedure that provides information for further clinical management. For patients who have thyroid nodules, US‐FNA reduces unnecessary surgeries and identifies individuals with a high risk of malignancy. The pathological outcome of US‐FNA was classified according to TBSRTC. Ultrasound and cytological findings can help differentiate noninvasive follicular thyroid neoplasms, with papillary‐like nuclear features, from the invasive encapsulated follicular variant of PTC.^30^ In addition, ultrasound findings are useful for cytopathologists to manage thyroid nodules with nondiagnostic or unsatisfactory thyroid FNA results.^31^ However, the risk of malignancy of Bethesda system III/IV nodules varies widely. Our study explored whether ultrasound characteristics of thyroid nodules facilitate clinicians interpreting the risk for malignancy of Bethesda system III/IV thyroid nodules to inform further therapies.

In our study, thyroid nodules with relatively TI‐RADS high scores received FNA, so total ROM of Bethesda system III/IV was 54.2%, which was significantly higher than those from Western practice,^29^, ^32^, ^33^, ^34^ demonstrating that TI‐RADS reduces the number of unnecessary thyroid nodule FNAs.^35^ Second, as we know differentiated thyroid carcinoma (DTC) accounts for more than 90% of all thyroid cancers and DTC is an indolent carcinoma, our practice of triaging patients with Bethesda system III/IV nodules is as follows: ≥TI‐RADS 4c with Bethesda system III/IV nodules were referred to surgery; ≤TI‐RADS 4a with Bethesda system III/IV nodules were referred for routine observation, with the exception of benign thyroid nodules, which were referred to surgery; for TI‐RADS 4b with Bethesda system III/IV nodules needing further discussion with the patients, possible advice included repeat FNA diagnostic surgery, routine observation, use of other imaging modalities, and so on based on patient preference.^36^, ^37^

In general, ultrasound features suspicious for malignancy include hypoechogenicity, irregular borders, being taller than wide, microcalcifications, and components of the nodules.^38^, ^39^ In our study, differences were observed in the rates of hypoechogenicity, irregular borders, being taller than wide, microcalcifications and components of nodules between benign and malignant groups. Reports indicated that being taller than wide, irregular margins and hypoechogenicity were all associated with malignancy in thyroid nodules, with irregular margins having the highest positive predictive value for malignancy.^38^ This observation was also reported by Maia et al^40^ in 80 patients surgically treated at a single center. In the present study, we observed irregular borders, hypoechogenicity, microcalcifications, being taller than wide, and components of the nodules all increased the risk for malignancy in thyroid nodules. This finding is in accordance with a study by Kure et al^41^, ^42^ Brito et al^43^ demonstrated that being taller than wide had the highest diagnostic OR for judging the malignancy of thyroid nodules compared to other graphic ultrasound features. In our study, a relationship between the dimension of being taller than wide and risk of cancer was observed.

Several studies have confirmed that the majority of malignant thyroid nodules are solid.^44^ However, the presence of microcalcifications in partial cystic lesions increase the risk of malignancy.^45^ Furthermore, a totally cystic lesion conveys a very low risk of malignancy and can be treated as benign disease.^43^ In our study, 93.8% (30/32) of patients had solitary nodules in the two malignancy subgroups, and 48.1% (13/27) had solitary nodules in the two benign subgroups. We found that solitary nodules increased the risk for malignancy in patients.

**Figure 4:**
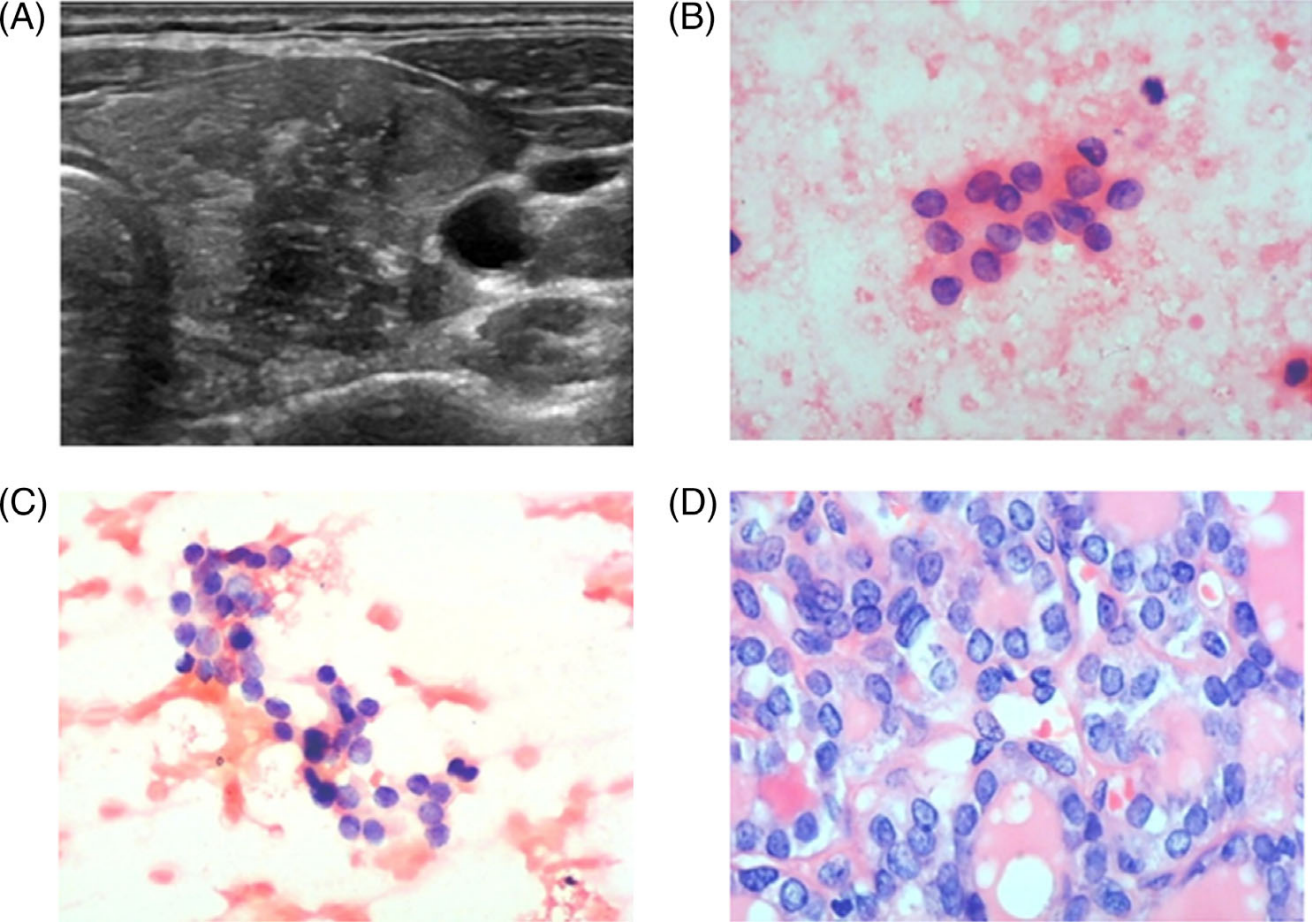
A case with AUS/FLU thyroid nodule. A, Imaging of the thyroid nodule. B,C, Results of FNA. D, Histopathology results. Figure 4A shows a solid thyroid nodule located in the left lobe of the thyroid. The nodule was hypoechoic with irregular border and microcalcifications (TI‐RADS 4c). Figure 4B,C show cytological atypia: focal nuclear changes and extensive but mild nuclear changes (×400). Hence, we diagnosed this thyroid nodule as atypia of undetermined significance or follicular lesion of undetermined significance (AUS/FLUS). Figure 4D shows histopathology of PTC for this thyroid nodule (×400). FNA, fine‐needle aspiration [Color figure can be viewed at wileyonlinelibrary.com]

**Figure 5:**
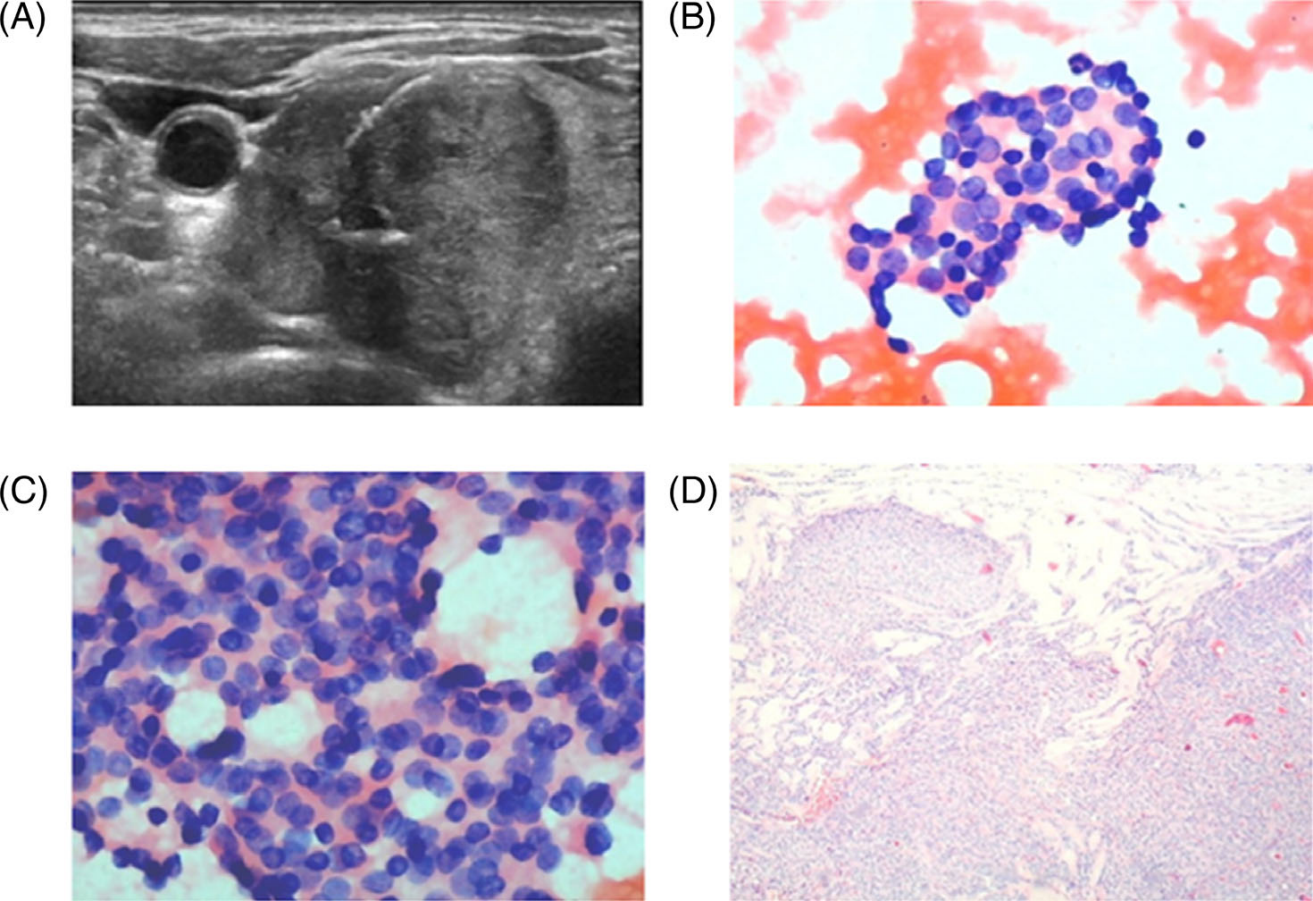
A case with FN/SFN thyroid nodule. A, Imaging of thyroid nodule. B,C, Results of FNA. D, Histopathology results. Figure 5A shows a solid thyroid nodule located in the right lobe of the thyroid. The nodule was hypoechoic with irregular border and macrocalcifications (TI‐RADS 4b). Figure 5B,C show mild nuclear changes (increased nuclear size, nuclear contour irregularity) without true papillae and intranuclear pseudoinclusions (×400). Hence, we diagnosed this thyroid nodule as follicular neoplasm or suspicious for a follicular neoplasm (FN/SFN). Figure 5D shows the histopathology was FTC for this thyroid nodule (×40) [Color figure can be viewed at wileyonlinelibrary.com]

The risk and incidence of malignancy in Bethesda system III/IV nodules varies widely. When nodules are diagnosed as Bethesda system III/IV, it is a challenge for clinicians to decide whether to recommend surgery, repeated US‐FNA or follow‐up. One study reported that repeated US‐FNA can be performed 6 months or more after initial AUS/FLUS.^46^ Rossi Met et al^47^ attempted to better define the management of Bethesda system III/IV nodules by introducing cytological subcategories, concluding that surgery may be applied in part of Bethesda system III and all Bethesda system IV nodules and that repeated FNA and follow‐up may be useful in part of Bethesda system III nodules. Vargas‐Salas et al^48^ addressed three critical topics for clinicians to facilitate improved decisions for treating thyroid nodules. They think molecular testing should be considered a public health measure, avoiding unnecessary surgical risk and cost. In the United States, molecular testing has become widely used to measure the risk of AUS/FLUS and FN/SFN. However, genetic tests have not been used in thyroid practice or included in the health insurance system in some areas of China and other countries. Meanwhile, when we emphasize molecular testing in the diagnosis of thyroid carcinoma, being mindful of expenses to the patient and health care costs to society were considered because it is equally important to avoid overuse of molecular tests.^49^ Another study concluded that clinical features, such as gender, nodule size, and age, should be considered in patients with undetermined thyroid nodule.^50^ However, in clinical practice, ultrasound features of thyroid nodules influence patient decisions, and final assessment of the nodules is based on various ultrasound features. Figures 4 and 5 show two patients with Bethesda system III/IV thyroid nodules. Figure 4A shows that a solid thyroid nodule was located in the left lobe of the thyroid. The nodule was a hypoechoic nodule with irregular border and microcalcifications (TI‐RADS 4c). FNA was conducted, and results revealed cytological atypia: focal nuclear changes with extensive but mild nuclear changes (Figure 4B, Figure 4C). Therefore, we diagnosed this thyroid nodule as AUS/FLUS. Finally, the patient decided to undergo surgery to resect this nodule, and the histopathology was PTC (Figure 4D). Figure 5 was a case of an FN/SFN thyroid nodule. Figure 5A shows a solid thyroid nodule located in the right lobe of the thyroid. The nodule was hypoechoic with irregular border and macrocalcifications (TI‐RADS 4b). FNA results showed mild nuclear changes (increased nuclear size, nuclear contour irregularity) without true papillae and intranuclear pseudoinclusions (Figure 5B,C). We diagnosed this thyroid nodule as FN/SFN and recommended surgery for this patient. Histopathology of this thyroid nodule was FTC (Figure 5D). In this study, we unveiled significant associations between solitary nodules, hypoechogenicity, irregular borders, being taller than wide, microcalcifications, and malignant nodules.

## 4 CONCLUSIONS

In conclusion, hypoechogenicity, irregular borders, being taller than wide, microcalcifications and solid nodules are ultrasound characteristics of malignant thyroid nodules. As the ROM of PTC was high, when any one of the five ultrasound features of malignancy was confirmed, repeated FNA is recommended to confirm or rule out PTC malignancy, even for Bethesda III/IV nodules. However, repeated FNA should be avoided when none of these ultrasound features are identified because a repeated FNA does not contribute to identifying non‐PTC malignancies, such as follicular thyroid carcinoma and poorly DTC.

## CONFLICT OF INTEREST

Fu Li, Denghua Pan, Yuquan Wu, Jinbo Peng, Qing Li, Xiaolong Gui, Wei Ma, Hong Yang, Yun He, Junqiang Chen declare that they have no conflict of interest.
